# Doing laboratory ethnography: reflections on method in scientific workplaces

**DOI:** 10.1177/1468794116678040

**Published:** 2017-04-13

**Authors:** Neil Stephens, Jamie Lewis

**Affiliations:** Brunel University London, UK; Cardiff University, UK

**Keywords:** animal models, cultured meat, laboratory ethnography, interaction order, methods, psychiatric genetics, robotic surgery, Science and Technology Studies, sensory ethnography, space

## Abstract

Laboratory ethnography extended the social scientist’s gaze into the day-to-day accomplishment of scientific practice. Here we reflect upon our own ethnographies of biomedical scientific workspaces to provoke methodological discussion on the doing of laboratory ethnography. What we provide is less a ‘how to’ guide and more a commentary on what to look for and what to look at. We draw upon our empirical research with stem cell laboratories and animal houses, teams producing robotic surgical tools, musicians sonifying data science, a psychiatric genetics laboratory, and scientists developing laboratory grown meat. We use these cases to example a set of potential ethnographic themes worthy of pursuit: science epistemics and the extended laboratory, the interaction order of scientific work, sensory realms and the rending of science as sensible, conferences as performative sites, and the spaces, places and temporalities of scientific work.

## Introduction

The 1970s saw the first ethnographies of scientific laboratories by early proponents of Science and Technology Studies (STS). This body of work set about demonstrating the local accomplishment and social construction of scientific knowledge. In this article, we reflect upon our own ethnographic practice in biomedical laboratories and the wider circuits of bioscientific work. Our aim is to provoke methodological discussion on how to approach the complex ethnography of laboratories. We do not propose here to provide any sort of ‘how to’ guide on access, data collection or writing. Instead, we hope to help attune potential laboratory ethnographers to some of the themes they may choose to collect data about and to analyse. In doing so we take lead from Atkinson (2015), in that we seek to identify ‘some generic phenomena, some generic social processes, that repay close and systematic attention’ (2015: 2). We do so through a set of ethnographic vignettes that report novel findings from our own laboratory ethnographies to explicate certain ways of thinking. Key to our perspective is the commitment to the interactionist tradition in ethnographic work that focuses upon interactive practice and the detailed observation of *how* scientific work is accomplished through interaction (Atkinson, 2015; Atkinson et al., 2008).

We begin with a brief introduction to the STS tradition of laboratory ethnography before outlining the specific field sites we have worked in that we draw upon later in the article. We then articulate a set of related themes through which the ethnographer may wish to situate their own fieldwork: the epistemic and the extended laboratory; the interaction order; sensory ethnography (both learnt and created); spaces, places and rhythms, and the performativity of conferences. Each of these accounts develops themes articulated in Atkinson (2015) by applying them in the context of STS through our own fieldwork sites. Importantly, by stressing the themes interrelated nature, we re-emphasise that good ethnography should never try to explain all that is observed through one explanatory mechanism, nor should it try to explain what is observed through every explanatory mechanism.

Knorr Cetina (1995) claims the first published laboratory study was Thrill (1972), although Lynch’s 1974 fieldwork was the first true to the STS tradition (Lynch, 1985a). She divides early STS work into (i) the controversy studies of Bloor (1976), Collins (1975) and Barnes (1977) that looked at how scientists assert legitimacy for their knowledge claims during disputes, and (ii) ‘the study of unfinished knowledge’ that closely inspects science in the making (Knorr Cetina, 1995: 140). This second group, led by Knorr (1977) herself, as well as Latour and Woolgar (1986 [1979]), Lynch (1985a) and Traweek (1988), most keenly employed the detailed ethnographic observation of day-to-day work to document how normal scientific knowledge is accomplished. This given, these authors still adopt different approaches to their laboratory studies, with Latour and Woolgar employing the ‘anthropologist of science’ (1986: 27) perspective through which mundane scientific practice is treated as a strange and alien culture, in contrast to Knorr’s (1977) focus on understanding the scientific work as the scientists do. Lynch, a student of Harold Garfinkel, pursued an ethnomethodological approach.

Hess (2001) labels all of these early studies ‘first generation’ STS ethnography, with their focus on ‘the way in which concerns with evidence and consistency were interwoven with situationally contingent events, local decision-making processes, … the interpretative flexibility of evidence … and other social or non-technical factors’ (2001: 234). He contrasts this with ‘second generation’ STS ethnographies that are orientated towards social problems, with a wider range of field sites beyond the physical working spaces of scientists to study lay groups, social movements, and political activities. The work we describe here remains within the working spaces of scientists, fitting Hess’s ‘first generation’, and continuing an ongoing strain of STS pursuing ethnography within laboratories (cf. Harrington, 2013; Sormani, 2014). Following Atkinson (2015), we argue the importance of fieldwork, in our case in scientific workspaces, as a rewarding and satisfying research endeavour, and we hope to inspire further work in this form. In what follows, we articulate what to observe and how to think about scientific workspaces through examples from our own work.

In the remainder of this article we draw upon five of Neil Stephens’ projects: (i) an ethnography with an interdisciplinary team of stem cell scientists, engineers, and physicists, who sought to produce novel imaging technologies for cell culturing; (ii) Neil’s current work in a laboratory developing robotic surgical tools to aid the implantation of cochlear implants; (ii) a yearlong project seeking to sonify the culturing practice of cell biologists in order that other scientists could better understand the protocol used through sound alone; (iv) a four-year ethnography of the UK Stem Cell Bank, an institution that holds all human embryonic stem cell lines in the UK and regulates their use (Stephens et al., 2008, 2011); and (v) an ongoing eight-year study of scientists who seek to tissue engineer meat for human consumption (O’Riordan et al., 2015; Stephens, 2013,). We also use two of Jamie Lewis’ ethnographies: (vi) a twelve-month study of a Huntington’s Disease research laboratory and animal house (Lewis and Atkinson, 2011; Lewis et al., 2013; Lewis et al., 2014); and (vii) a laboratory conducting work in the genetics of neuropsychiatric conditions. While our ethnographies have been conducted independently, we have also written comparative work on Jamie’s Huntington’s disease (HD) study and Neil’s work in the UK Stem Cell Bank (Stephens et al., 2013). These two projects also involved Paul Atkinson as a team member.

We report previously unpublished findings from these ethnographic sites as a set of vignettes to provoke reflection on what laboratory ethnography can do, and how ethnographers can go about doing it.

## Ethnography of the epistemic

The classic theme of laboratory ethnography is that scientific knowledge production is an accomplishment and, as such, the epistemic is situated and cultural (Hess, 2001; Knorr Cetina, 1995). While we believe ethnographic work can fruitfully move beyond focusing upon this alone, it remains vital that this fundamental focus upon the epistemic is not lost, and that new ethnographies continue to explore knowledge production as situated within new technical domains and new political contexts. The epistemic component of laboratory ethnography was foregrounded in Jamie’s work in a laboratory testing the efficacy of cells grafts to alleviate symptoms of HD. The laboratory functioned through a set of relations between four spaces: the ‘dry’ laboratory, where mathematical analysis is conducted; the ‘wet’ laboratory, where experiments are conducted, and in this case, the brains of rodents are analysed; the ‘clinic’, where patients were enrolled into clinical trials; and the ‘animal house’, where rodents are kept, cared for, and prepared for experimental procedure.

Central to Jamie’s analysis is the production and use of what are known as ‘animal models’: animals - frequently rodents – that have been subject to physical interventions and discursive and documentary regimes to configure them as models of human disease states. Jamie was told that in the case of HD the disease is reduced to a limited set of phenomena – for example, gait, attention span, balance - that are rendered observable in the animal models. These models are induced using three main strategies: inactivating an existing gene (knock-out rodents), inserting a foreign gene (transgenic rodents), or making a non-genetic change (lesioned rodents), with each strategy intended to mimic the observable cognitive and motor deficits of patients with HD. As Jamie has shown elsewhere, typically establishing such equivalence involves accepting *good-enough* solutions based upon the *judgements* and *skillsets* that inform them (Lewis et al., 2013). Here, we analyse an instance when Jamie observed these judgements and skillsets in action in the animal house while watching the training of mice for a balance test.

A technician was busy with four small cages containing a mixture of normal and transgenic grey mice. The technician explained that the next day she would be running the test ‘for real’ during which she would not know which ones were transgenic. For now, however, it was fine for her to know as she focused upon training the mice to walk up a raised thin ramp leading to a fixed box at the top. The ramp was tapered, getting thinner as it went, allowing her to test the hypothesis that transgenic mice would struggle with the ascent as their feet, mirroring some of the behaviours associated with HD, slipped off the beam. She explained to Jamie that initially the bar was not raised, but the experiment did not work, because the mice simply remained at the end they were placed. She suggested raising the ramp to encourage the mice to move. As each mouse was placed with their tail facing the box (their heads facing away), they were encouraged to turn and scuttle up the beam into the house where they would be rewarded with a treat. The technician tested all twenty mice and timed their ascents, with some moving with agility and others more clumsily. Jamie asked the technician whether those that struggled to walk up the beam were the transgenic mice. She smiled saying ‘yes, well, for the most part’, but explained that some mice are simply not that good at walking up the bar. Presumably, some mice, like some humans, are more comfortable with heights than others. The time data of all the ascents were sent to the PI to run some statistics, with the technician explaining that the PI is ‘brilliant at finding patterns in numbers’.

The role of technicians is often under-represented in the scientific process (Shapin, 1989). It was clear, though, that the technician’s *skills* and *judgements* were paramount to modelling aspects of HD in the laboratory. There are clearly multiple reasons why a mouse may or may not walk up a beam swiftly with some of these nothing to do with any genetic manipulation. As someone who has studied, cared for, and trained the mice, the technician was able to provide further context about the mouse’s performance, gaining an insight into its personality, its walking ability, its likes and dislikes. This tacit knowledge, gleaned from the day-to-day labour and skilled handling of animals, is *constitutive of the epistemic work* of HD. In concert with the statistical and observational work of the other laboratory spaces, the animal house, as part of the extended laboratory, contributes to the accomplishment of scientific knowledge, which is made not discovered, through situated and culturally informed practice. This is a well-made point in ethnographic STS, but still one to which ethnographers should remain attentive.

## The interaction order of scientific meetings

Central to the work of any ethnographer is the study of interaction. Team meetings are key sites of the *interaction order* (Goffman, 1983); they are local and intimate *encounters* that deserve detailed ethnographic inspection. To achieve this, the laboratory ethnographer can draw upon the insights of conversation analysis, and analysis of the embodied and material aspects of performance, without necessarily engaging in fully-fledged conversation analysis transcript work (Atkinson, 2015). Neil’s study with the interdisciplinary group of stem cell scientists, physicists, engineers, and chemists explored this in terms of the boundary-work and performative display of disciplinary identity during the project’s day long group meetings. Importantly, the account not only argues that interdisciplinary work is performed, but details *how* this performance occurs, and *how* asymmetrical relationships within it are embedded within a mundane interactional grammar.

The meetings Neil observed were intended to bring together the entire consortium (normally based in three separate campuses across two cities) to update each other on progress, ensure all team members were getting the support they needed, check where the group were in relation to their milestones, and to foster an interdisciplinary environment. They occurred every three months, with each hosted by a different disciplinary group, in their respective departmental building. Such mobility was intended to capture the sharing of disciplinary spaces and the non-hierarchical environment sought by the team. Despite different geographical settings, the meetings retained a standardised patterning in their temporal, spatial, and interactional structuring. While the rooms used took various shapes - from long and narrow, to square - the team members frequently formatted the furniture around a rectangle of tables, allowing each attendee to face each other for discussion or face a speaking position and projector for presentations.

The meetings also retained a formal structure starting with a greeting by the Principal Investigator (PI) who would remind the group of the milestones and the project timeline. This was followed by two or three forty-minute talks from postdocs, followed by lunch and then another two forty-minute talks, before a closing PI raises once more the milestones set for the next three months. An informal structure also quickly arose featuring a layered use of language based around disciplinary expertise. During presentations, or in conversations addressing the whole group, speakers would typically soften the most esoteric terminology in an attempt to develop a mutually accessible repertoire. Often during formal presentations, or during group discussion, subsets of more specialised discourses would occur concurrently. The physicists, the engineers, or the cell biologists, would have hushed conversations employing specialist terminology accompanied by gestures such as moving the hands close to the face, and lower volumes of talk, to minimise intrusion of this disciplinary talk into the group space.

This layering of a shared group repertoire and closed specialist repertoire, the differing material and embodied practices that support them, and the occasional interruptions that disrupt it, are both performative of, and responsive to, the *doing* of the interaction order of this interdisciplinary meeting space. However, Neil notes it was clear that one specialist terminology – that of cell culturing – held a privileged position above other disciplines, in that the esoteric cell biological terminology interrupted and dominated the shared discussion space more frequently, and for longer durations, than any other phrasing. Group esoteric physics discussions, for example, were held between few speakers, rarely lasted long, and typically handed topic control back to the shared group through repair work like ‘we’ll discuss this later’, accompanied by hand gestures identifying a small subset of team members. In contrast, group esoteric cell biological discussions could be extended, with turns exchanged between multiple speakers with expertise in cell culturing unhindered by interruptions from others, or a sense that such language excluded non-biologists. This asymmetric intrusion and turn-taking captures a moral order within the team meeting; in this case, prioritising cell biology, but potentially favouring other disciplines when found as a structural component of interaction in other settings. The work of the ethnographer is to note not only its occurrence, but also the mundane interactional features upon which its performance is based.

## Sensory ethnography: learning what’s sensible

STS is increasingly attentive to multimodality, with long standing interests in the visual (Lynch, 1985b; Stephens and Ruivenkamp, 2016; Vertesi, 2015) recently being complemented with a growing literature on the audible (Mody, 2005; Pinch and Bijsterveld, 2012), and new works focusing upon touch, taste, and smell (Alač, 2011; Greiffenhagen, 2014; Nishizaka, 2011). A focus upon the sensory remains in ethnographic work, with some reminding us to retain the long-standing tradition of sensory attentiveness (Atkinson, 2015; Atkinson et al., 2008; Stoller, 1989, 1997); while others promote the use of sensory ethnography attuned to emplacement (Pink, 2015).

Neil has conducted two laboratory ethnographies that focus on how epistemic and technical innovation is rendered *sensible* through interaction, employing the dual meaning of sensible as both *perceivable* and *comprehensible*. The first is an example rendering the imperceptible as sensible, and is based on an account of his very first encounter in the robotic surgery laboratory.

The robotic surgical tool laboratory developed a range of devices, but the main focus of their work at the time of the ethnography was a drill designed to cut through human tissue (skin, fat, muscle) with high precision and the capacity to predict what tissue lay just ahead of its current position. The primary purpose the laboratory developed it for was to aid in operations to insert cochlear implants into the inner ear while reducing damage to the hearing tissues.

Within the first ten minutes of being by the laboratory bench, Neil was shown the robotic surgical drill. The silver handheld device was rectangular; around 16cm in length, with a small drill bit extending a few cm from one end, with coiled wires running from the other end to a laptop perched above more computer hardware. It had already been explained to him that, based upon existing modelling of tissue, the drill and its software could predict what tissue type lay just ahead of it as it drilled into the body basing its calculations on force and torque readings from the drill bit itself. In use, the drill could be held by a surgeon pushing it into the inner ear and towards the sensitive and important hearing tissue of the endosteal membrane. The one automated function of the drill was to stop, based on its prediction of the immediate tissue environment, just as it touched the membrane, but without damaging it. This precision, Neil was told, would be impossible for a surgeon unaided. The measurements were imperceptible to humans because they were so small.

Over the course of the ethnography, it became clear that the laboratory had a number of strategies for rendering this imperceptible precision sensible. On day one, Neil participated in what had become something of a welcoming ceremony for visitors to the laboratory, and a key guided practice in experiencing the drill’s sensitivity: the act of drilling into an egg. The lead scientist retrieved an egg from the laboratory fridge, and demonstrated how the tool could drill through the shell of the egg but would stop when it touched the membrane that separated shell from the egg white without damaging the membrane. Neil was then given the opportunity to try himself. Pressing the drill, as it drilled into the eggshell, the soft vibrations of the tool were perceivable, as was the drilling sound. Neil had never drilled into an egg before, and knowing how much pressure to apply was not obvious. The scientist encouraged him to push harder as the shell powdered and a small hole less than 2mm wide was created. Holding the drill straight was no simple task for a complete beginner, and Neil had several aborted attempts as the drill slipped, leaving half-finished holes in the egg’s surface. The moment of the first successful drill was marked by the predictive capacity of the drill itself, as its algorithms determined the membrane had been reached, and the drill, and its accompanying noise and vibrations, ceased.

Removing the drill, the scientist gestured toward the successful hole as evidence of the tool’s precision. Neil, again demonstrating no native competency, was not sure what he should see and how this confirmed anything. To him, the successful hole, and the holes from the multiple aborted attempts, all looked the same. The scientist assured him they were different, and took the egg to a microscope so the holes could be better inspected. After a few moments of balancing the egg, finding the holes, and focusing the microscope, the scientist invited Neil to view the magnified image, which again to Neil appeared as a set of white circles. Explaining the image, the scientist provided an account of how the white of the membrane was different to the white of the shell. Neil surmised that, since this membrane tone of white extended continuously across the hole, that the membrane was, as suggested, undamaged and that the drilling was successful.

This account then demonstrates just one strategy of how the drill’s imperceptible sensitivity is rendered sensible, both perceivable and comprehensible. This was accomplished through a choreographed demonstration based upon multi-sensory experience. The sensory alignments of feeling and hearing the drill, seeing the white circles (by naked eye and microscope), and having their interpretation narrated, was performative of the drills capabilities. It invoked a particular interpretation of a sensory realm, which was taught and enacted through interaction.

## Sensory ethnography: creative sensory alignments

The second of Neil’s ethnographies on the sensory in scientific work explored what those involved in the project called ‘the Stem Cell Orchestra’. Here, Neil was both participant in, and ethnographer of, the research team. The core interdisciplinary group consisted of two musicians, two biologists, Neil, and a bioinformatician. Bioinformatics is a specialism that applies computing to biology, often in the context of ‘big data’ (Lewis and Bartlett, 2013). This project was based upon developing the team’s in-house bioinformatics software ‘*ProtocolNavigato*r’. The programme was designed to replace cell biologists’ lab books with an electronic version that could record the procedure of each experimental protocol, share it with other scientists, and display the protocol via a novel flowchart based graphical interface. The challenge of the Stem Cell Orchestra was to add sonification to the software so that protocols could be translated into an audible representation as opposed to a visual one, and to articulate why the audible version improved scientific practice. Like the robotic egg surgery example, this account again highlights the sensory alignments required in sharing interpretations of an epistemically productive sensory realm. However, the two cases differ in that while the egg example performed a *pre-scripted* set of actions, interpretations, and sensitising practices, the Stem Cell Orchestra case examples the *creative production* of a sensory realm and the interactional encounters invoking the alignments that render their interpretation sensible.

Cell culturing research is usually conducted at a hooded cell culture bench. Cells are seeded into wells in a plate, expanded within the well, harvested out of the well, washed, and seeded again into new wells. During this ongoing reseeding process, drugs, dyes or growth factors can be added to some of the wells (while others are left untouched as comparative controls) to record what difference the added materials make. Experiments can last several weeks, and typically involve short bursts of reseeding activity separated by long periods of incubation during which the cells remain untouched. In this case, the laboratory was working with cancer cells and adding the dye DRAQ7. As an ethnographer, Neil observed and audio recorded team meetings. As a contributor to the project from an STS perspective, he articulated the challenges of recording, sonifying, and then sharing an essentially tacit cell culture practice.

To help sonify the DRAQ7 protocol the musicians would first have to understand how it worked. This involved lengthy discussions at team meetings and visits to the cell culture laboratory to watch and video record the experiment being conducted. They observed pipetting, felt the warmth of the incubator, and knelt down to hear the clicking of the fridge as it cooled down to the appropriate temperature. They asked questions, joked about the complexity, and articulated the rhythms of seeding, harvesting, and incubating. Back in team meetings, they would sit with synthesisers, pre-recorded sounds, or improvised vocalisations to suggest soundscapes and earcons that capture both the essence, and some epistemically valuable content, relating to the cell culture work (*cf.* Supper, 2015 on how sonification experts do similar in conferences).

Over the course of a year, a sonic repertoire of rhythms, melodies, and harmonic ratios were developed that led up to a defining moment in the first feedback session, when cell culturers from outside the core group joined a project meeting to hear the results, and discuss whether they could make sense of them. During this meeting, a novel sensory realm was articulated, through playing sounds, watching an accompanying visual animation, conversation, and the embodied work of gesturing, humming, and tapping out rhythms. Through these processes of *in situ* negotiation, the group agreed that some key components of cell culturing practice were recognisable, and thus sensible, in the sonification, while others were said to be missing the mark. For example, the rich sustained string sound was agreed to capture the warmth of incubation, and the cymbal recognised as demarking the passage of time, although the exact amount of time remained unclear. Through re-listening, suggestive comments, and invocations of prior experiences of laboratory work or musical environments, the group agreed on the partial emergence of a sensible sensory realm; one in which new alignments between cymbal rhythms and hours of incubating, and piano chords and cell culture well plates, made the sonification of bioinformatics cell culture protocol software a perceivable and comprehendible practice.

As both the robotic surgery and the Stem Cell Orchestra examples show, laboratories are sensory spaces, replete with both phenomena to be sensed and technologies that sense. They are also sites of interaction and shared sense-making around the proper interpretation and enactment of these sensory alignments. Laboratory ethnography offers the opportunity to document not only the work these sensory realms achieve, but also how they are accomplished through interaction. By attuning their analysis to a multi-modal sensuousness the ethnographer can work to further extend the reach of STS beyond the visual into the interconnections of sensory epistemics.

## Spaces, places and rhythms

Ethnographic fieldwork is not conducted in vacuum, it is situated, and we should consider the choreography of social life in these spaces (Atkinson, 2015). Space bannisters – simultaneously constrains and guides – social life and work, shaping the organisation of everyday life *in situ.* The scientific workspace is no different in this regard to any other place of work. Laboratory ethnography therefore benefits from attending to the rhythms of day-to-day work, the movements and flows of matter and matters, transformations and transitions, and boundaries and barriers.

Scientific work is accomplished, furnished and anchored in the laboratory. These come in different shapes and sizes: some are large and spacious; others small and confined, some are busy and heavily populated, others quiet and conspicuous by the absence of workers. Many are gated communities; others are linked closely to clinics and hospitals. Some are distant from the city and prying eyes (for example, the UK Stem Cell Bank, Stephens et al., 2008) and some are transient spaces that pop-up as portable packages (Lewis et al., 2014).

To reflect the importance of place and space in biomedical work, Neil, with colleagues, coined the term *performative architecture* to describe how the organisation and architecture of buildings reflects and frames the social actions within it (Stephens et al., 2008). The UK Stem Cell Bank building is therefore simultaneously symbolical and practical, performing aspects of cleanliness, sterility and safety. These ideas develop upon Thrift (2006) who comments on the ‘radical redesign of scientific space, reflected in the construction of numerous new performative buildings’ (2006: 292). New life science buildings such as these, he argues, are designed not only to intentionally produce intense social action between scientists, but are also built to reflect the state and status of bioscientific innovation.

Jamie’s ethnographic work articulates another, different, example. He studied a group working on psychiatric genetics that recently left their old laboratory space in an early 2000s building attached to a working hospital, to move to a purpose-built, state-of-the-art construction, similar to those described by Thrift. Architecturally, the laboratory’s new building has clean lines and razor sharp edges symbolising the cutting-edge work conducted inside. The glass exterior is not only modern but enacts the transparency and accountability of the work inside. The cathedral like atrium on the bottom floor has a large white space that performs openness, welcoming visitors and publics, yet the revolving access door means that guests cannot simply slip in unaccounted. There is also a coffee shop, a large lecture theatre, two training rooms and a hub for postgraduate research activity.

The building has a *rhythm*; weekday mornings and afternoons are the domain of the researcher and support staff, public events are confined to late afternoons/early evenings, and only the deadline or experimental protocol-tied researcher and the occasional security guard are seen at night or weekend. The building also has multiple purposes: it is home to a hospital research clinic as well as an out-of-sight animal house.

The different functions of the building’s spaces are contested. The open plan offices on floors 1, 2 and 3 are shared between the laboratory and another research institute. They work to provide durability to the social networks, symbolising collaborative and interdisciplinary research by creating a critical mass of expertise. Big science, we are told, requires scientists coming together co-located in these innovative incubators (Thrift, 2006). However here the symbolic and the functional come into conflict, as the symbolic openness of glassless windows in the shared atrium allows the noise of multiple users to reverberate through the offices spaces upstairs. This is escalated during late afternoon and early evening public events downstairs, in which film showings, choirs, or school visits create an overlapping sensory realm of differing interpretations of what constitutes reasonable noise. Staff complain about the volume inhibiting their concentration, through informal conversations, emails, and formal requests for action. Some seek alternative working spaces. This contestment overlays the hierarchies of the broader institution. Senior researchers and academics are provided with their own, quieter, offices, mid-career academics with shared offices, and early career researchers are located in the hubbub of the open plan area. When the flows of noise lead to the underuse of the building, the spare office space is offered to PhD students from other departments, for whom space restrictions mean they have no desk in their own disciplinary spaces. In this way, the building becomes a different type of interdisciplinary space, more performative of the frustrations of resource limitations and design blunders than innovative capacity.

Spaces, places and temporalities are locally occasioned, locally ordered and locally negotiated. Architectures, as physical things, give durability to the functional and symbolic work imagined by their creators, but they retain some flexibility in the reconfiguration of their physical and symbolic ordering, which occurs through the interactions of people in, around, and through these spaces (*cf.* Gieryn, 2002). They can be contested and reconfigured, be that through the simple rearranging of furniture in the ‘three monthly meetings’ or the move to install glazing in open plan areas. Ethnographers should be attentive to these spaces, places and temporalities. In studying their choreography and interactional forms orchestrated through them we see that place is always in the making (Hurdley, 2015), often sedimented, but rarely settled; it is an accomplishment worthy of study.

## Conferences as performative sites

Conferences are a key set of encounters in which scientific fields come together to interact, yet they remain under-analysed in STS (González-Santos and Dimond, 2015; Supper, 2015). Claims and counter-claims are made, bonds are confirmed or broken, and new ideas are presented as part of the pathway to rendering them legitimate and knowable. Both presenting papers, and being an audience member, are types of performance that require analysis of how they are put together (Atkinson, 2015; Dimond et al., 2015). Eating spaces, hotel lobbies, and mingling opportunities are also important sites of scientific work.

Conferences form part of the calendar, frequently on an annual basis, and, beyond just presentations, host award ceremonies, early career researcher support opportunities, journal editorial board meetings, and academic society AGMs. In established disciplines, the conference works to reinforce a field’s identity through performances of progress that retain continuity with the past. They have a grammar, an interaction order, which is recognisable across multiple disciplines.

Here, we focus on Neil’s research on a new, emergent, field: ‘cultured’ or ‘in vitro’ meat. The technology involves the application of tissue engineering (a typically biomedical practice) to producing meat. Cells are taken from an animal (typically an agricultural animal, but any animal is possible) and expanded under laboratory conditions into larger quantities of muscle tissue that is then presented as food. Laboratory research in the field began just after the millennium, beginning with as few as two laboratories active and today perhaps as many as ten. The field had its highest profile moment in 2013 when the world’s first laboratory grown hamburger was cooked and tasted at a London press conference intended to publicise the potential environmental, ethical, and health benefits of a meat made ‘outside of a cow’ (O’Riordan et al., 2016). The field remains small, and spreads across the university and private sector. Every so often, a new laboratory will display its latest prototype product accompanied by promissory accounts of how close it is to commercialisation (often tempered by issues about how much future funding could be made available). The field remains optimistic that progress is being made and that initial high end products will be delivered in the near future.

Neil’s work studies the ontological ambiguity of this technology to explore how the field seeks to establish a stable categorisation of tissue engineered muscle as food and, in some instances, as meat as we know it today (Stephens, 2013). Here, we explore the role of conferences as key sites of this work as participants operate to produce a shared narrative among themselves, and perform that narrative beyond the field.

Between 2008 and 2015, four key conference-like international events occurred in the field. The second, the ‘European Science Foundation Exploratory workshop in In Vitro Meat’ was held in Gothenburg in 2011, with Neil in attendance. The event was genuinely a meeting place; many of the scientists present had never met (or in many instances even emailed) the scientists from other countries. The workshop’s introductory character was felt across the event through those initial handshakes, the presentations on preliminary findings, and the closing press conference in which the technology was introduced to the Swedish press.

The event featured an explicit conversation among attendees on the naming of their technology and, by implication, their field. The merits of many names were articulated, but the main contenders were the existing ‘in vitro meat’ and the keenly advocated for (by a small portion of attendees) ‘cultured meat’. More than ‘a name’ were at stake in this discussion, as the very status of the technology and the participants’ research programmes were implicated. The term ‘in vitro’ remained the preference for some participants as it was seen as the scientifically accurate phrase. That said, it was recognised that the term could alienate some people in a way less likely with ‘cultured’, which benefitted from the combined meanings of ‘cell culture’, ‘fermented’ and ‘artistic or classy’ (*cf.* Datar, 2015). ‘Cultured’ was embraced by some who deemed it more appealing to potential consumers, and was accepted by others because the cell-culturing element meant it remained within a scientific repertoire. However, it was clear many in the room would not accept a term that distanced the science altogether; many present identified themselves as basic science researchers, not marketers, and that any name would need to be of the scientific realm, with a subsequent renaming of the technology to be led by a different set of professionals (perhaps in public relations) in a future moment once the technology shifted from research to commerce. As such, through the exchange on naming, the field were defining themselves, the proper category of work they engaged in, and the status of the technology and the tissue itself.

All conferences do work: they site interaction and perform and enact moments of science-in-the-making. For the ethnographer, they provide valuable research opportunities whatever the status of the field. In studying *what* is accomplished and *how* accomplishment happens at conferences the laboratory ethnographer can only enrichen their understanding of scientific practice and improve their studies.

## Conclusion

Ethnography should be attentive to the diversity of practices, orders, and frames of interpretation enacted in the contexts studied, while still producing a focused account that does not try to explain everything through everything (Atkinson et al., 2008). This is true of the laboratory as it is of any other field site. In this article, we have provided a set of ethnographic vignettes intended to provoke reflection upon some of this diversity, while retaining a focus upon *how* scientific work is accomplished through interaction, set in and through spaces, places and rhythms, including the extended laboratory and the conference. We have no problem with Hess’ second wave of STS ethnography moving beyond the laboratory and encourage this work, although we do not report on it directly here. Instead, we have reasserted the classic STS focus on the epistemic context of knowledge in the making and controversial science, while emphasising the interaction order and the sensory realm. We have done so to provoke laboratory ethnographers to consider the structural forms that routinely shape scientific practice in their own work. In this regard, the article is a call for the continued reinvigoration of laboratory ethnography. We hope people will be confident in their ethnography, attentive to diversity, and continue to produce ethnographically grounded STS.
